# Placenta Prævia

**Published:** 1866-04

**Authors:** L. P. Garrison

**Affiliations:** Cedar Town, Georgia


### *Placenta Prœvia.*—By L. P. Garrison, M. D., of Cedar Town, Georgia.

The advised plans of procedure in the management of pregnancy with this anomalous attachment of the placenta, differ so entirely from each other, that the young practitioner must necessarily be greatly embarrassed in the conduct of a labor, and in the previous treatment, which is generally necessary for some months before the conclusion of gestation. When practicable, our efforts should be directed to the safety of both mother and child, and the dictates of humanity, as well as our moral and professional obligations, would urge us to that course; but, when both must necessarily perish without such interference as will endanger the child, it is of course justifiable to make the sacrifice.

The following case exhibits that troublesome result, hemorrhage, to an unusually alarming extent, requiring, in the opinion of the writer, premature delivery for the woman's safety:

Was called, May 1st, to Mrs. S—-, on account of uterine hemorrhage. Found her about eight months advanced in pregnancy, and the hemorrhage, though profuse, controllable by the usual means of cold applications and the internal use of acetate of lead. By the occasional use of lead, the bleeding was kept in abeyance for about a week, when I was again called in haste, owing to the excessive discharge. The difficulty was only temporarily arrested, returning at intervals of an hour or two, and in twenty-four hours from the time I arrived, became so excessive and alarming, that the production of premature labor was determined upon. For this purpose the ordinary doses of ergot were given at proper intervals, and persisted in for a reasonable time, without producing any perceptible contraction of the uterus. Muriatic acid was then resorted to, in the dose of three drops every fifteen minutes, and in less than three-quarters of an hour from the administration of the first portion, active labor came on. By passing the finger into the os, I readily detached the placenta for a short distance, but finding difficulty, at the time, in separating it entirely, forcibly pierced its center with the finger, making extensive laceration. The liquor amnii was immediately discharged, and the head, advancing rapidly, was soon engaged in the superior strait, pressing firmly upon the torn edges of the placenta.

The labor was concluded safely to the mother, without turning or further interference, in about two hours from the commencement of labor.

